# Evaluation of the Influenza A Replicon for Transient Expression of Recombinant Proteins in Mammalian Cells

**DOI:** 10.1371/journal.pone.0013265

**Published:** 2010-10-11

**Authors:** Florian Krammer, Jens Pontiller, Christopher Tauer, Dieter Palmberger, Andreas Maccani, Martina Baumann, Reingard Grabherr

**Affiliations:** Department of Biotechnology, Vienna Institute of BioTechnology, University of Natural Resources and Applied Life Sciences, Vienna, Austria; Hallym University, Republic of Korea

## Abstract

Recombinant protein expression in mammalian cells has become a very important technique over the last twenty years. It is mainly used for production of complex proteins for biopharmaceutical applications. Transient recombinant protein expression is a possible strategy to produce high quality material for preclinical trials within days. Viral replicon based expression systems have been established over the years and are ideal for transient protein expression. In this study we describe the evaluation of an influenza A replicon for the expression of recombinant proteins. We investigated transfection and expression levels in HEK-293 cells with EGFP and firefly luciferase as reporter proteins. Furthermore, we studied the influence of different influenza non-coding regions and temperature optima for protein expression as well. Additionally, we exploited the viral replication machinery for the expression of an antiviral protein, the human monoclonal anti-HIV-gp41 antibody 3D6. Finally we could demonstrate that the expression of a single secreted protein, an antibody light chain, by the influenza replicon, resulted in fivefold higher expression levels compared to the usually used CMV promoter based expression. We emphasize that the influenza A replicon system is feasible for high level expression of complex proteins in mammalian cells.

## Introduction

Expression of therapeutic proteins in mammalian cells is a growing field in biotechnology. Although yields can be optimized by the choice of cell line, expression vector, and adequate process design, there still is a need for new expression systems as well as promoter elements [1], [2]. A great number of viral replicons and attenuated or replication deficient viruses for heterologues gene expression derived from RNA viruses have been developed during the last twenty years. Most of them were designed to be used as vehicles for gene transfer and recombinant protein expression in mammalian cells or as vaccines. These include the+sense single stranded (+ss) RNA virus members of the *Togaviridae* like Semliki Forest virus, equine encephalitis virus, rubella virus or Sindbis virus and members of the *Flaviviridae* like Kunjin virus as well as – sense single stranded (−ss) RNA virus members of the *Rhabdoviridae*, *Paramyxoviridae* and *Orthomyxoviridae* [3], [4], [5], [6], [7], [8]. The system which has shown the greatest potential for recombinant gene expression is beyond doubt the Semliki Forest virus replicon. A review from 1992 which reports the progress on alphavirus replicons for recombinant gene expression also suggests to use an influenza A replicon for the same purpose [9]. Over the years, influenza virus reverse genetics techniques developed rapidly [10] and culminated in the invention of bidirectional plasmid based systems [11]. Although these systems are far away from being easy to handle they provide a perfect tool to evaluate the influenza A replicon for the expression of recombinant proteins. To date, a small number of proteins, namely the chloramphenicol transferase (CAT), the green fluorescent protein (GFP), and the firefly luciferase have been expressed from influenza replicons for matters of transfection control for influenza virus reverse genetics and better understanding of the influenza replication machinery [12], [13], [14]. Additionally, GFP, a mycobacterial epitope and interleukin-2 have been expressed by an viable active influenza A virus from an altered NS genomic segment [8], [15], [16]. Here we describe the expression of the human anti-HIV-gp41 antibody 3D6 [17] by the replicon of the influenza A virus strain A/Hiroshima/52/2005 (H3N2). Additionally, we expressed the light chain individually, evaluated the effect of using different non-coding regions (NCRs) on the expression level and investigated the influence of temperature on the influenza replicon activity.

## Results

### Transfection efficiency

**Figure 1:**
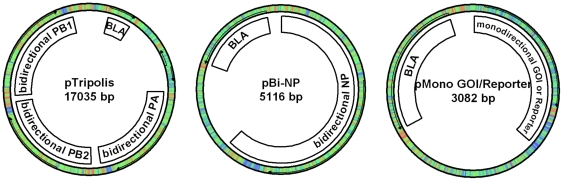
Schematic drawings of plasmids used to generate the influenza replicon based expression system. pTripolis and pBi-NP generate the influenza replicon (mRNA/protein and vRNA) whereas pMono plasmids with various genes of interest or reporter genes drive transcription of vRNA.

**Figure 2:**
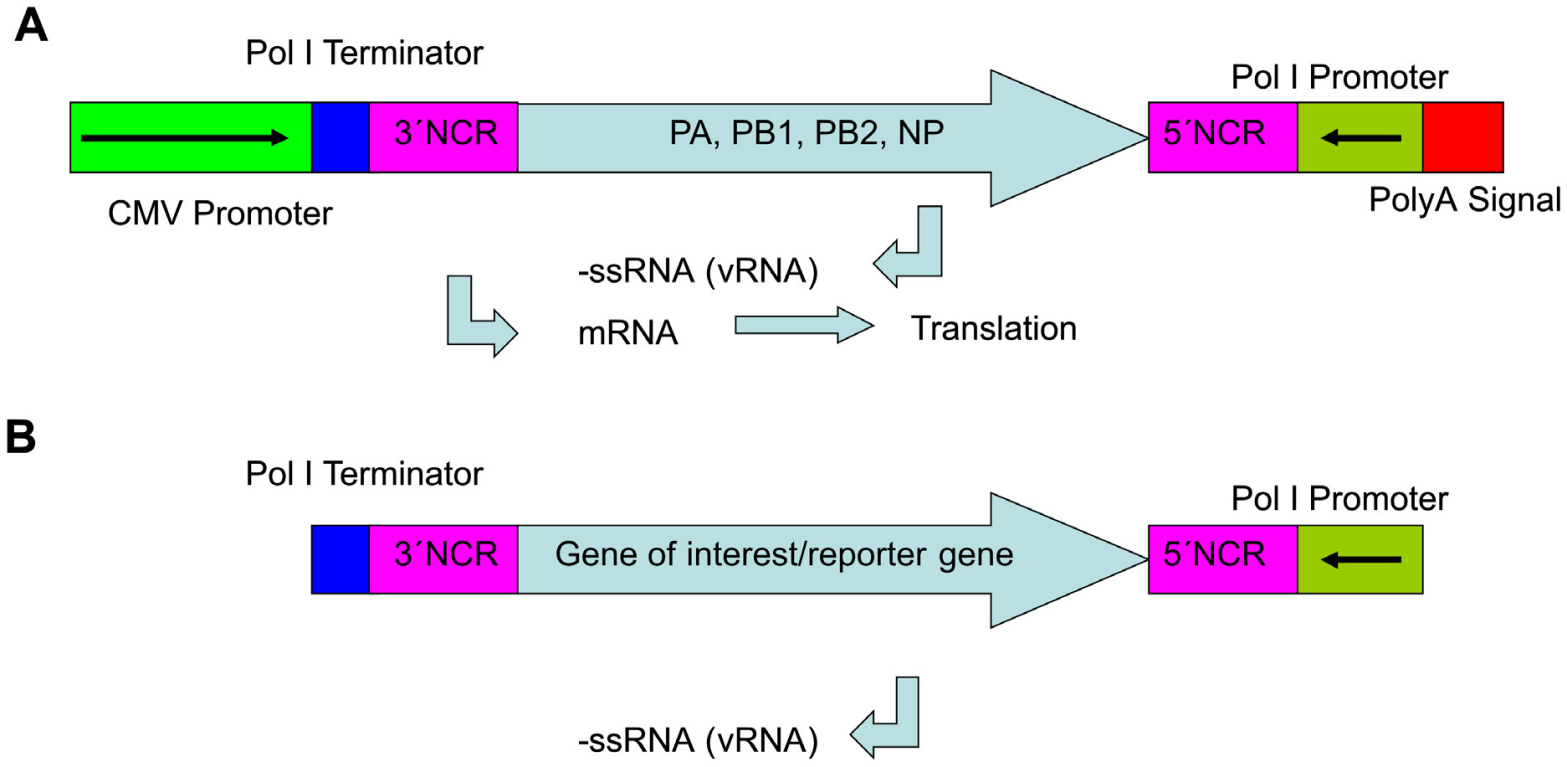
Schemata of bidirectional and monodirectional transcription cassettes. Bidirectional cassettes (A) were used for generation of the influenza replicon. Monodirectional cassettes (B) were used to generate vRNA of reporters or genes of interest.

**Figure 3:**
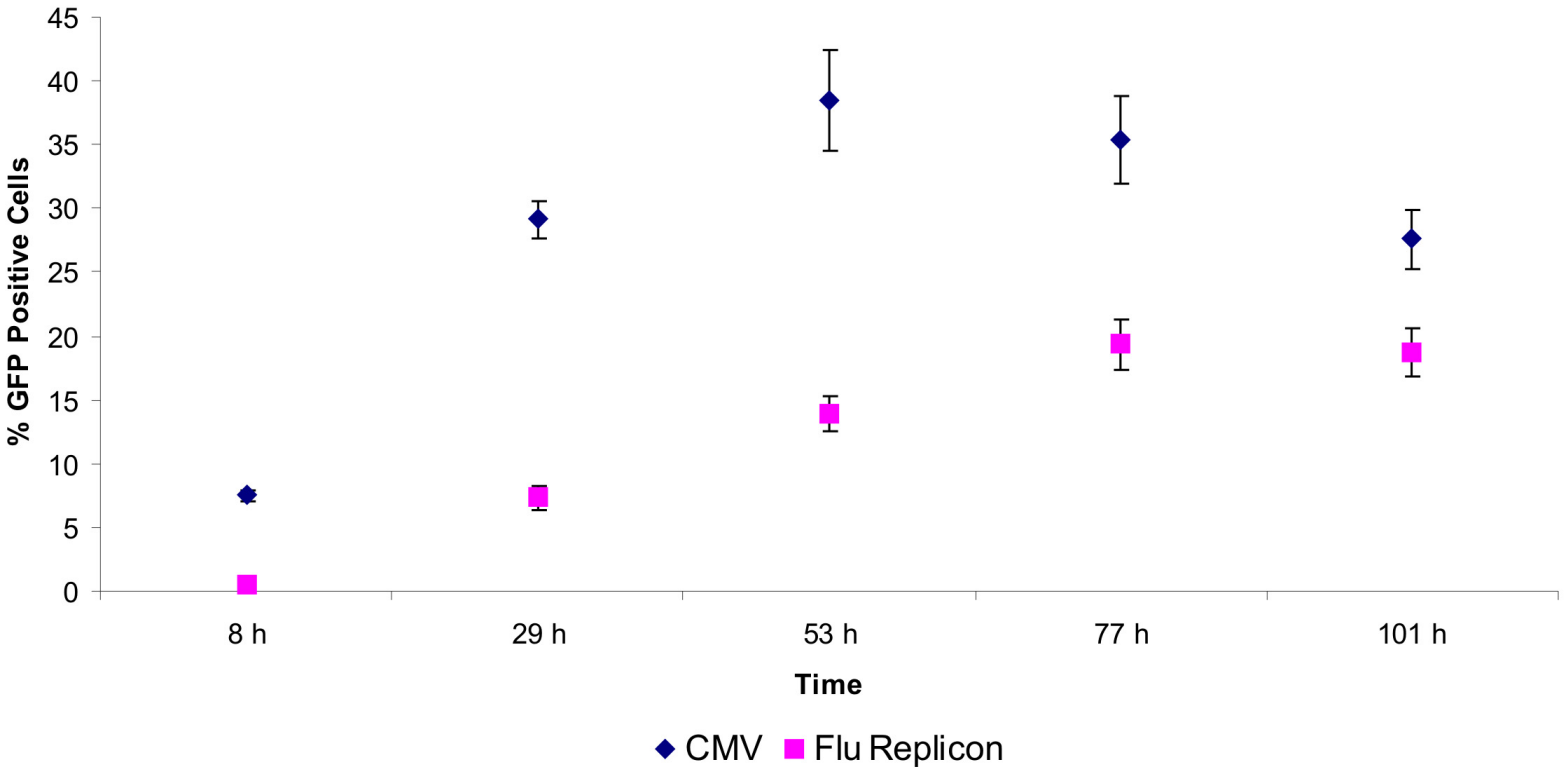
Transfection efficiency time course. HEK-293 cells either tranfected with pEGFP-N1 (CMV) or with the influenza replicon and pMono-EGFP (Flu Replicon) were monitored over a period of 101 hours by FACS analysis. Data represents arithmetic mean values and standard deviation.

**Figure 4:**
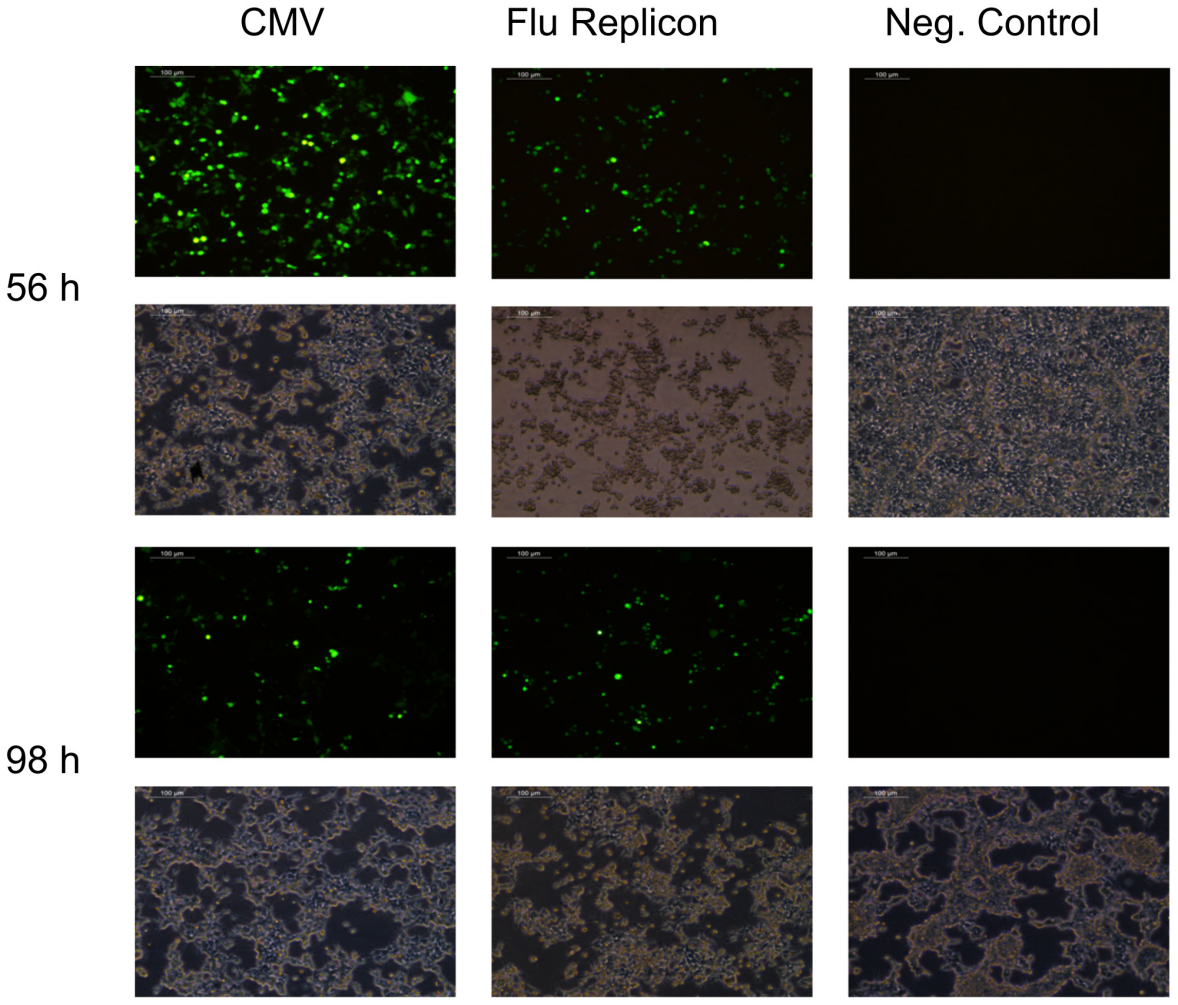
EGFP expression. HEK-293 cells were either tranfected with pEGFP-N1 (CMV) or with the influenza replicon and pMono-EGFP (Flu Replicon). Pictures were taken 56 and 98 hours post transfection. Non-transfected cells were used as negative control (Neg. Control).

The efficiency of transfecting mammalian cells can be influenced by a number of factors like the choice of method, condition of the cells, purity of DNA, plasmid size and number of different ones to be delivered per cell. The influenza replicon that we have chosen to evaluate is based on two plasmids (17035 bp and 5116 bp) which encode viral RNA (vRNA) and proteins which are necessary for the formation of the influenza replicon (Figure 1). Plasmids which provide anti-sense RNA (analogous to vRNA) of a reporter gene or gene of interest (Figure 1 and 2) were co-transfected. We compared this system to a single plasmid with human cytomegalovirus immediate-early promoter (CMV) driven expression in terms of transfection efficiency. As a reporter gene for this particular experiment we chose EGFP and analyses were performed by FACS. HEK-293 cells were transfected with pEGFP-N1 (CMV) or pTripolis (bidirectional transcription of influenza polymerases PB1, PB2 and PA), pBi-NP (bidirectional transcription of influenza NP) and pMono-EGFP (Figure 1, Table 1). The vector pMono-EGFP provides antisense transcription of the EGFP reading frame driven by a human RNA polymerase I dependent promoter fragment (below referred to as RNA polymerase I promoter), the EGFP gene is flanked by 5′ and 3′ non-coding regions from the influenza NS genomic segment. The percentage of EGFP expressing cells was determined over a period of 101 hours (Figure 3 and 4). Eight hours post transfection (h.p.t.) approximately 7.5% of the pEGFP-N1 transfected cells were EGFP positive as compared to a very low percentage of positive cells found in the populations transfected by the replicon system. The percentage of EGFP expressing cells in the population of pEGFP-N1 transfectants increased to 29.2% percent after 29 h.p.t. and peaked with 38.5% at 53 h.p.t. The cells transfected by the replicon system showed a delayed peak of 19.4% positive cells at 77 h.p.t. . In contrast to the pEGFP-N1 (CMV) driven control the percentage of EGFP positive cells in the replicon transfected fraction decreased very slowly after the peak was reached.

**Table 1:** Plasmids and plasmid amounts used for various experiments.

Experiments	EGFP CMV	EGFP Replicon	Luciferase CMV	Luciferase Replicon NCR NS	Luciferase SV40	AB CMV 1∶1	AB Replicon 1∶1	AB CMV 10∶1	AB Replicon 10∶1	LC CMV	LC Replicon	Luciferase Replicon NCR M	Luciferase Replicon NCR PB1	negative control (HEK)
Plasmids														
pEGFP-N1	9 µg													
pcDNA-luc			9 µg											
pGL3-control					9 µg									
pRC-LC						4.5 µg		0.45 µg		9 µg				
pRC-HC						4.5 µg		4.5 µg						
pTripolis		3 µg		3 µg			3 µg		3 µg		3 µg	3 µg	3 µg	
pBi-NP		3 µg		3 µg			3 µg		3 µg		3 µg	3 µg	3 µg	3µg
pMono-EGFP		3 µg												
pMono-lucNS				3 µg										3µg
pMono-LC							3 µg		0.3 µg		3 µg			
pMono-HC							3 µg		3 µg					
pMono-lucM												3 µg		
pMono-lucPB1													3 µg	
pRL			1 µg	1 µg	1 µg							1 µg	1 µg	1µg
**Total Amount**	9 µg	9 µg	10 µg	10 µg	10 µg	9 µg	12 µg	4.95 µg	9.3 µg	9 µg	9µg	10 µg	10 µg	7µg

pEGFP-N1, pcDNA luc, pRC-LC and pRC-HC carry the CMV immediate-early promoter, pGL3-control and pRL carry SV40 promoter elements, pTripolis and pBi-NP drive bidirectional transcription (mRNA and vRNA) of the influenza polymerases and the NP protein. pMono-EGFP, pMono-lucNS, pMono-LC, pMono-HC, pMono-lucM and pMono-lucPB1 drive monodirectional (vRNA) transcription of the gene of interest. AB stands for antibody, LC stands for light chain experiments.

**Figure 5:**
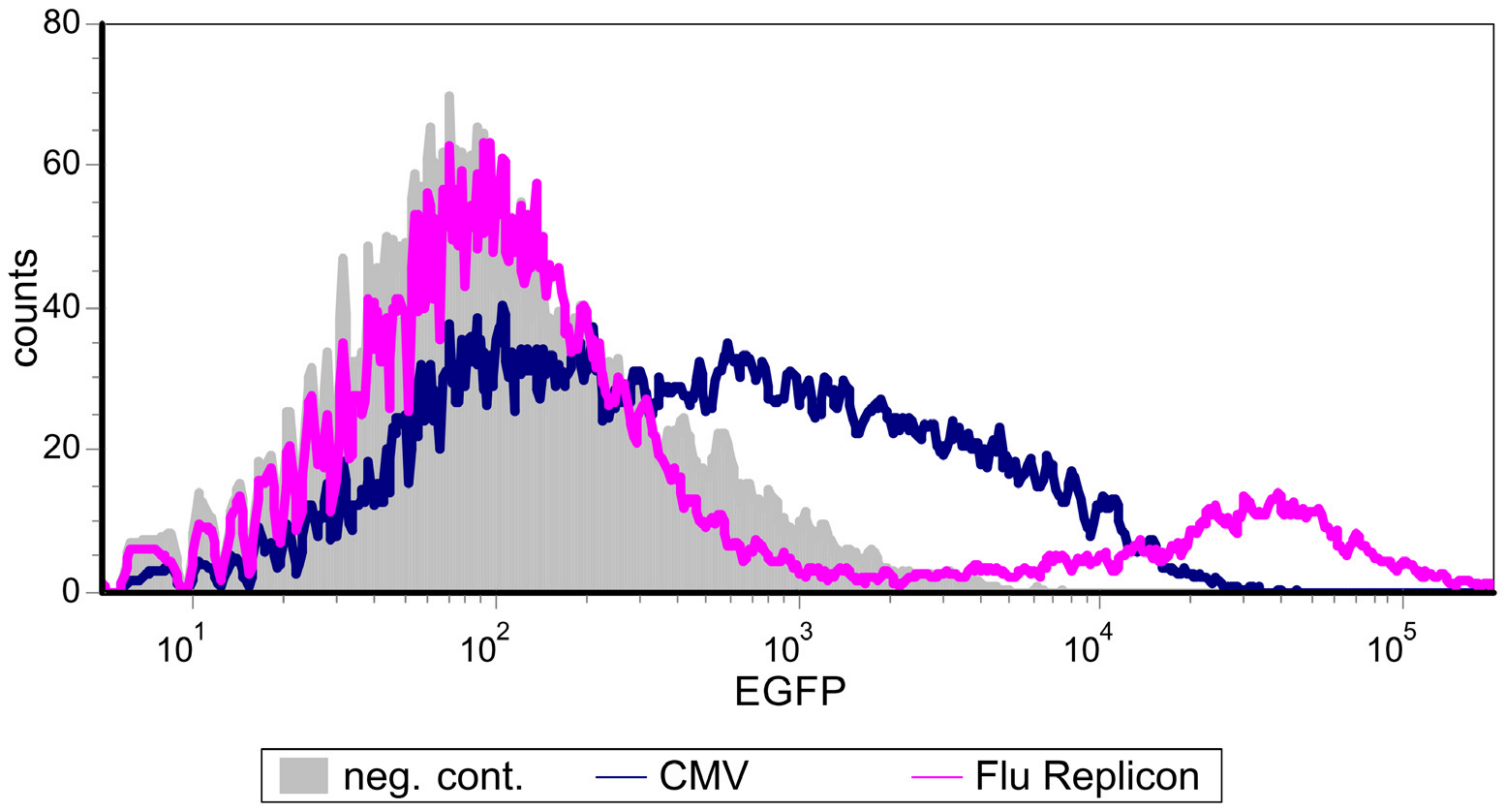
FACS analysis of cells transfected with either pEGFP-N1 (CMV) or the influenza replicon system and pMono-EGFP (Flu Replicon) 77 hours post transfection. Cells transfected with the replicon system show two very distinct populations in the forward light scatter (EGFP), one completely negative, the other highly positive. pEGFP-N1 driven EGFP expressions shows a broad distribution spanning from completely negative to moderate and high expressing cells.

Another interesting phenomenon was that cells transfected with the replicon system showed two distinct populations (Figure 5). One population was EGFP negative while the other population expressed high levels of EGFP. Almost no cells with low or moderate EGFP expression were found in between these two peaks. In contrast, the pEGFP-N1 transfected cells showed a broad distribution spanning from completely negative to moderate and high expressing cells.

### Analysis of expression level by luciferase assay

**Figure 6:**
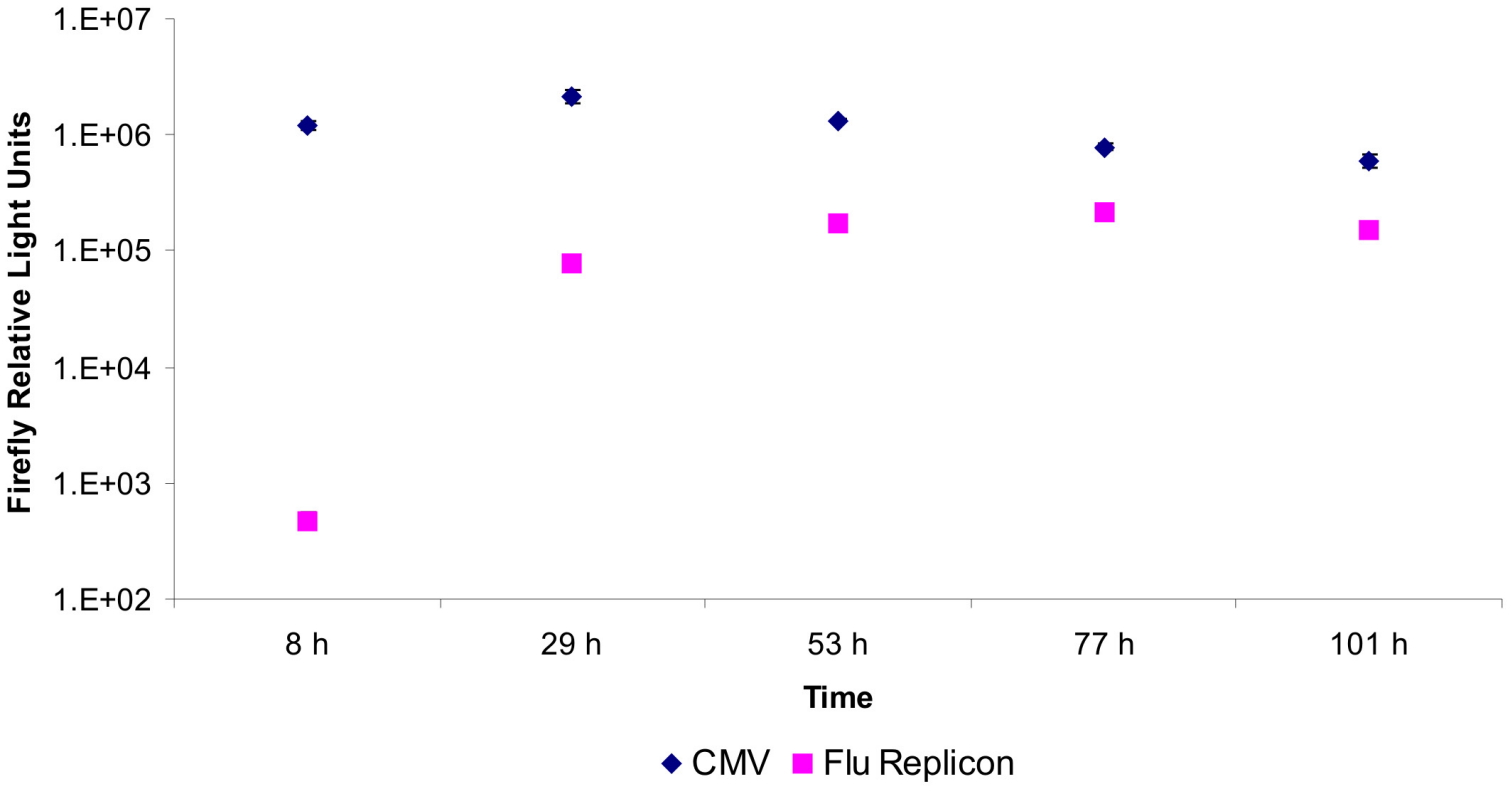
Time course of firefly luciferase expression. HEK-293 cells either tranfected with pcDNA-luc (CMV) or with the influenza replicon and pMono-lucNS (Flu Replicon) were monitored over a period of 101 hours by luciferase assay. Data represents arithmetic mean values and standard deviation.

Based on our results from the FACS experiments we speculated that although the percentage of positive cells in populations transfected with the replicon system was lower than in the pEGFP-N1 transfected populations, however, the overall expression level may still be higher due to a higher percentage of strongly expressing cells. In order to quantify expression levels in more detail we repeated the experiment using the firefly luciferase instead of EGFP as the reporter. Non-transfected HEK-293 cells were used as negative control in all luciferase experiments. Additionally we used a second negative control which was transfected with pMono-lucNS and pBi-NP but not with the pTripolis plasmid to verify that the vRNA alone is not translated. The luciferase expression level was monitored over a period of 101 hours, including a pcDNA-luc (CMV) driven firefly luciferase as positive control. Observed data resembled the previous experiment to some degree, however the activity peak for the pcDNA-luc control was detected at 29 h.p.t. in contrast to 53 h.p.t. in the previous experiment (Figure 6). The replicon system showed the same time course as observed in the FACS experiment (Figure 6). However, the expression level of the replicon system measured by luciferase activity was unexpectedly low, between 13% (53 h. p.t.) and 27% (77 h.p.t.) of the level of the pcDNA-luc driven control.

**Figure 7:**
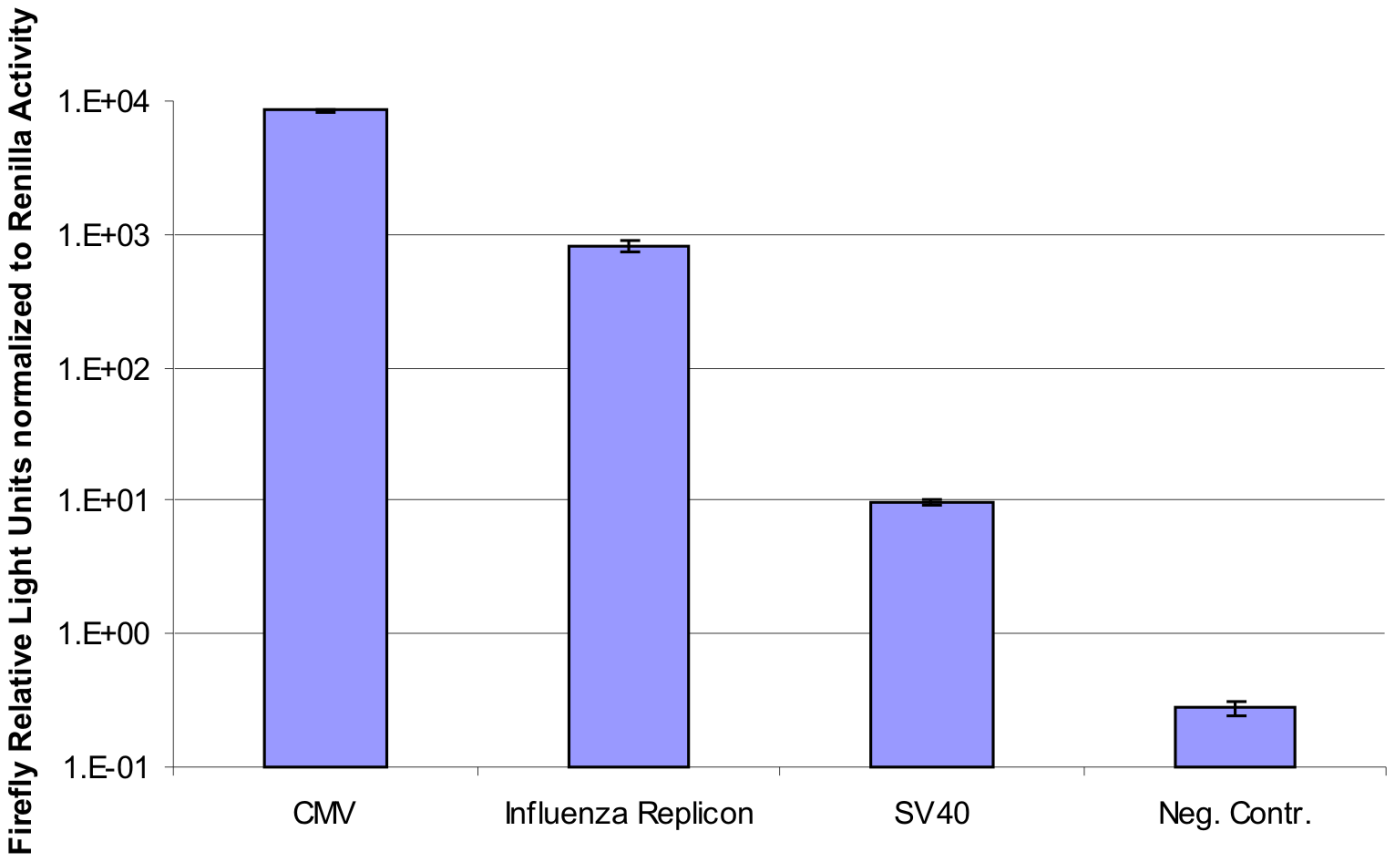
Comparison of firefly luciferase expression driven by CMV, SV40 or by the influenza replicon. HEK-293 cells were either tranfected with pcDNA-luc (CMV), pGL3-control (SV40) or with the influenza replicon and pMono-lucNS (Influenza Replicon) or with pMono-lucNS and pBi-NP only (Neg. Contr.). Additionally, pRL, coding for *Renilla* luciferase, was cotransfected in all cases. Cells were harvested 48 hours post transfection and subjected to luciferase assay. Firefly values have been normalized to *Renilla* values. Data represents arithmetic mean values and standard deviation.

We further decided to compare the influenza replicon system to CMV and SV40 promoter (SV40) driven expression in HEK-293 cells. In order to be able to normalize firefly values to an internal standard we co-transfected pRL DNA which codes for *Renilla* luciferase driven by an SV40 promoter. Normalization was applicable in all experiments except in time course experiments described above because the *Renilla* expression is not stable over time. HEK-293 cells were transfected with pTripolis, pBi-NP, pMono-lucNS and pRL or pcDNA-luc and pRL or pGL3-control (firefly luciferase under control of SV40 promoter) and pRL or pBi-NP, pMono-lucNS and pRL (negative control) (Table 1). Cells were harvested 48 hours post transfection and were subjected to the luciferase assay. The expression value of the replicon system (normalized value of 817.7) showed approximately one tenth of the activity of the CMV driven construct (normalized value of 8479.9) but approximately 100 fold activity of the SV40 driven construct (normalized value of 9.8). The negative control transfected with pBi-NP and pMono-lucNS showed no activity at all (firefly luciferase: 128±5 RLU, *Renilla* luciferase: 466.8±37.7 RLU, normalized value of 0.3) (Figure 7).

**Figure 8:**
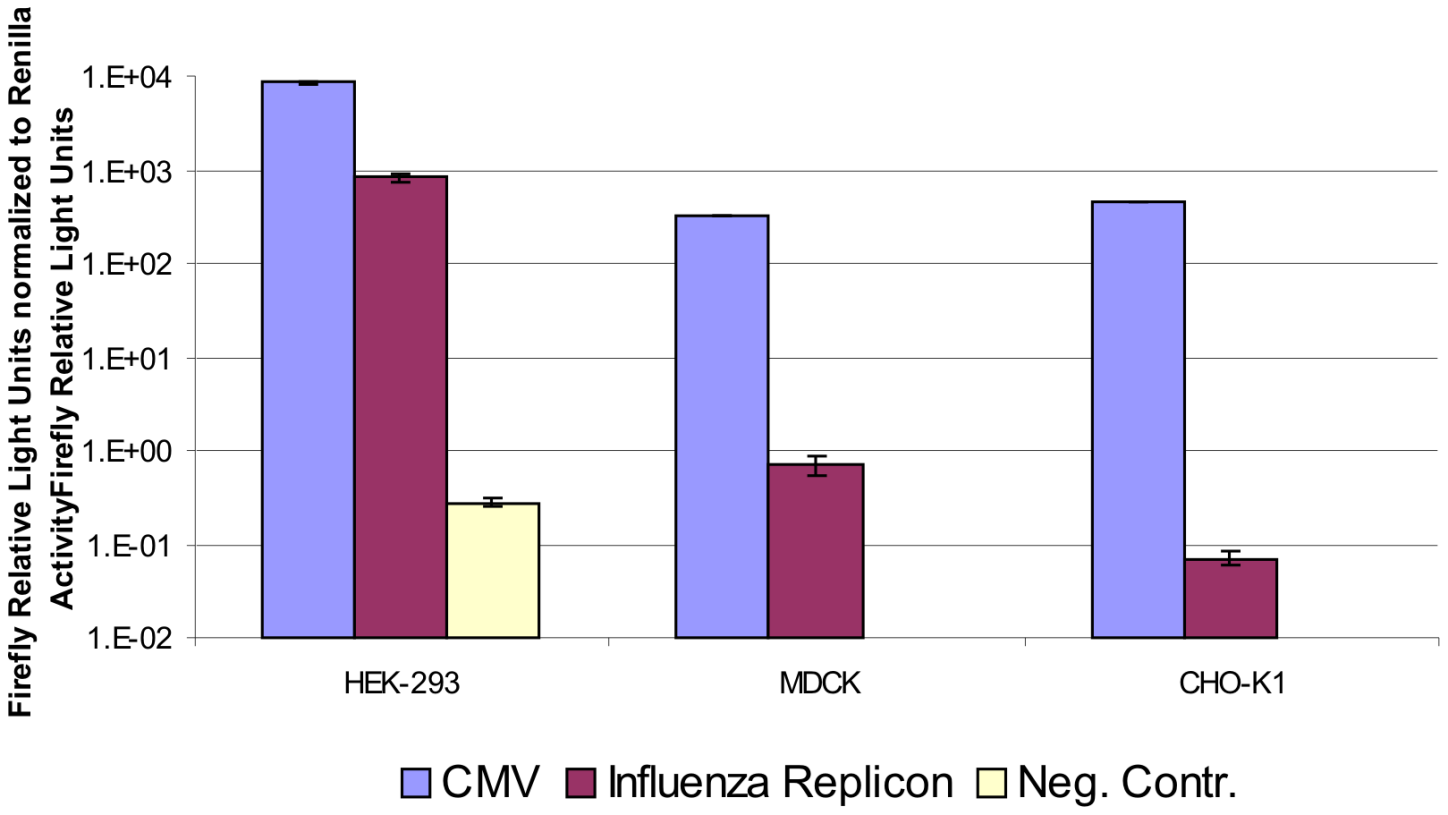
Activity of the influenza replicon in a human, a canine and a rodent cell line. HEK -293, MDCK or CHO-K1 cells were either tranfected with pcDNA-luc (CMV) or with the influenza replicon and pMono-lucNS (Influenza Replicon). The HEK-293 negative control has been transfected with pMono-lucNS and pBi-NP only. Additionally, pRL, coding for *Renilla* luciferase, was cotransfected in all cases. In case of MDCK and CHO-K1, untransfected cells have been used as negative controls and can therefore not be normalized, absolute values can be found in Results section. Cells were harvested 48 hours post transfection and subjected to luciferase assay. Data represents arithmetic mean values and standard deviation.

The human RNA polymerase I promoter used to drive vRNA transcription in our experiments is known to work in a very restricted number of cell lines derived from primates. However, we wanted to verify this restriction by comparing the activity of the replicon system in the human HEK-293 cell line to activity in a canine (MDCK) and in a rodent (CHO-K1) cell line. Cells were transfected with pTripolis, pBi-NP, pMono-lucNS and pRL or pcDNA-luc and pRL. As expected, luciferase activity was detected in HEK-293 cells (firefly luciferase: 137040±17119 RLU, *Renilla* luciferase: 168.3±7.1 RLU) but not in the MDCK (firefly luciferase: 89±21 RLU, *Renilla* luciferase: 125.8±9.6 RLU) and CHO-K1 (firefly luciferase: 94±4.4 RLU, *Renilla* luciferase: 1349.3±194.0 RLU) cells which showed values minimally higher than the HEK-293 negative control (firefly luciferase: 128±5 RLU, *Renilla* luciferase: 466.8±37.7 RLU) or the untransfected controls for MDCK (firefly luciferase: 72±0 RLU) and CHO-K1 (firefly luciferase: 77±7 RLU) (Figure 8).

### Temperature dependence of the influenza A virus replicon

**Figure 9:**
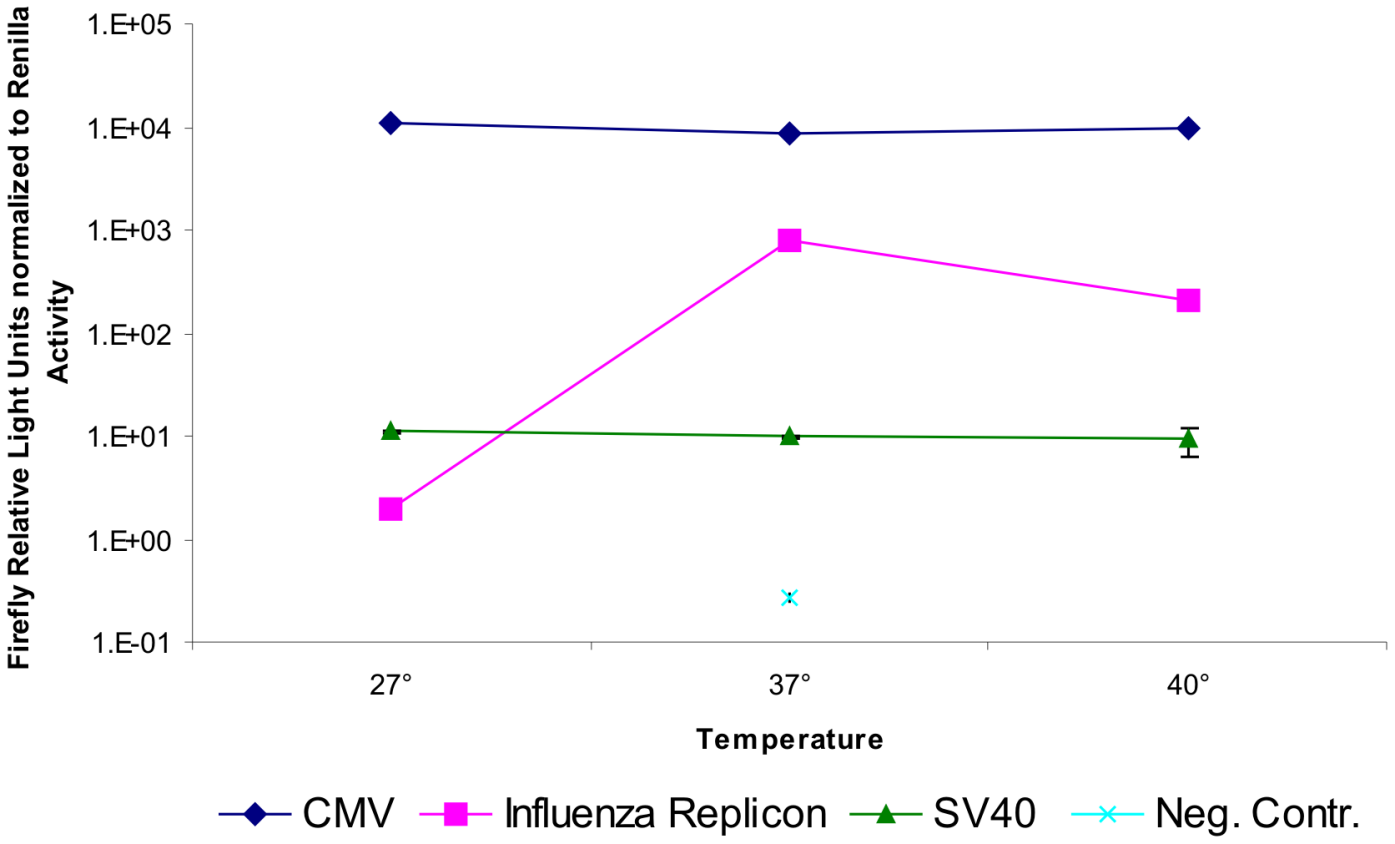
Temperature dependence of the influenza replicon. HEK-293 cells were either tranfected with pcDNA-luc (CMV), pGL3-control (SV40) or with the influenza replicon and pMono-lucNS (Flu Replicon) or with pMono-lucNS and pBi-NP only (Neg. Contr.). Additionally, pRL, coding for *Renilla* luciferase, was cotransfected in all cases. Four hours post transfection cells were transferred to 27°C or 40°C or remained at 37°C. Cells were harvested 48 hours post transfection and were subjected to luciferase assay. Firefly values have been normalized to *Renilla* values. Data represents arithmetic mean values and standard deviation.

As a next step, we decided to analyze the activity of the replicon expression systems at different temperatures, namely 27°C, 37°C, and 40°C. HEK-293 cells were transfected with pTripolis, pBi-NP, pMono-lucNS and pRL or pcDNA-luc and pRL or pGL3-control (firefly luciferase under control of SV40 promoter) and pRL. Cells were allowed to settle at 37°C for 4 hours and were then transferred to incubators heated to 27°C or 40°C or were further incubated at 37°C. Cells were harvested 48 hours post transfection and subjected to luciferase assay. Again, firefly luciferase values were normalized to *Renilla* activity. Interestingly, the cells transfected with the replicon system which were incubated at 27°C showed no activity at all. Activity at 40°C was reduced to approximately 25% of the activity at the optimal temperature of 37°C indicating a strong temperature sensitivity of the influenza viral polymerases (Figure 9).

### Influence of the influenza genomic non-coding regions on the expression level

**Figure 10:**
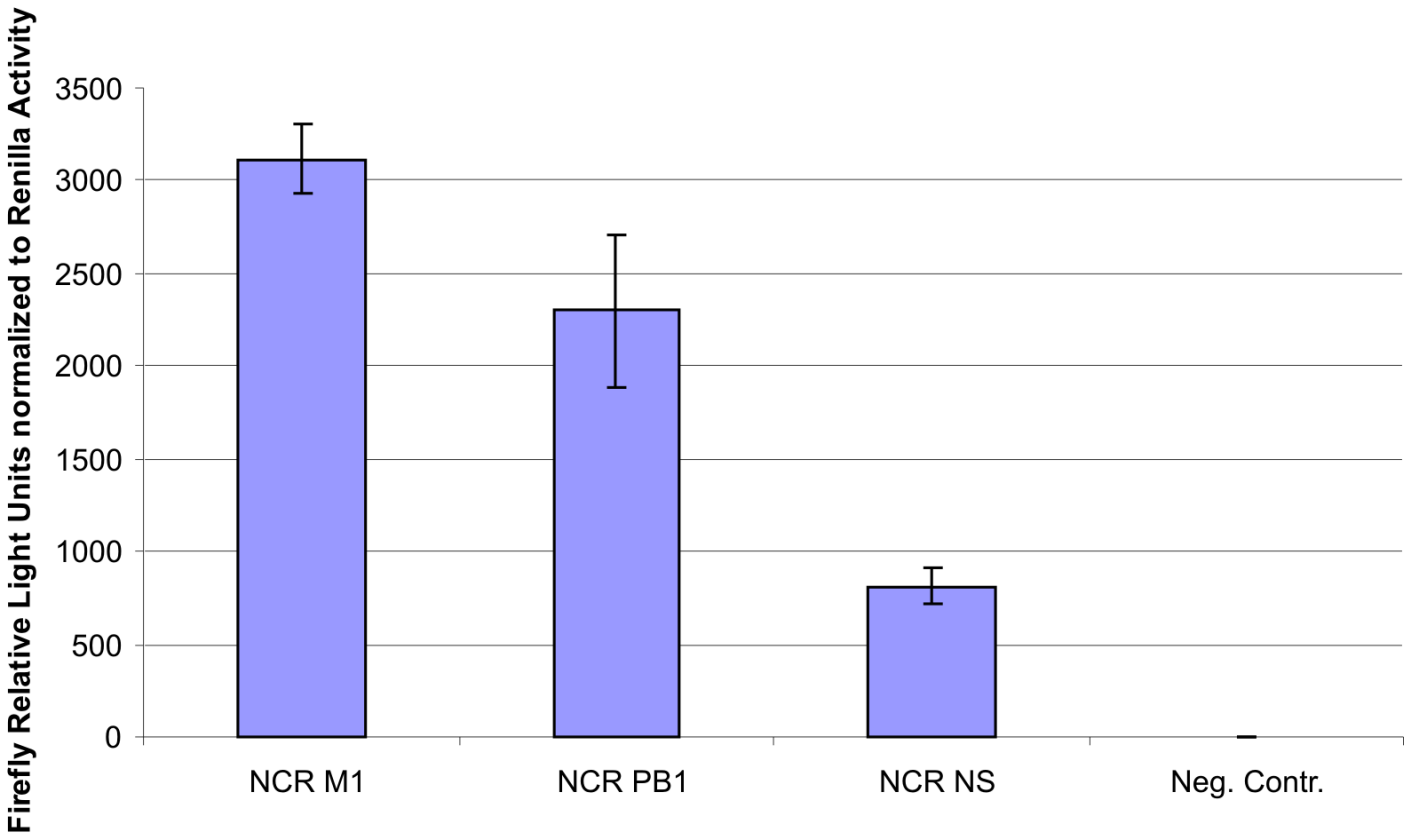
Influence of different NCRs on the expression level . HEK-293 cells were tranfected with the influenza replicon and either pMono-lucNS (NCR NS) or pMono-lucM (NCR M) or pMono-lucPB1 (NCR PB1) or with pMono-lucNS and pBi-NP only (Neg. Contr.). Additionally, pRL, coding for *Renilla* luciferase, was cotransfected in all cases. Cells were harvested 48 hours post transfection and subjected to luciferase assay. Firefly values have been normalized to *Renilla* values. Data represents arithmetic mean values and standard deviation.

Recently, it was shown that the composition of the 5′ and 3′ non-coding regions (NCR) of influenza vRNA has strong influence on influenza gene expression [18], [19], [20], [21]. Therefore, we decided to test NCRs from different genomic segments in the luciferase assay. In the previously described experiments we used a construct which contained the NCRs of the NS genomic segment of A/Hiroshima/52/2005. The NS genomic segment codes for two proteins, the non-structural protein 1 (NS1) which is not incorporated into viral particles, and the nuclear export protein (NEP). Additionally, we chose to fuse NCRs from the PB1 genomic segment and the M genomic segment to our reporter gene. The two proteins encoded from the M genomic segments (M1 and M2) are incorporated into the viral particles where M1 is present in high abundance. The PB1 protein is part of the trimeric polymerase complex of influenza A and eight copies of this protein can be found per particle. In order to analyse the influence of the different NCRs on the expression level we transfected HEK-293 cells with either pTripolis, pBi-NP, pMono-lucNS and pRL or pTripolis, pBi-NP, pMono-lucPB1 and pRL or pTripolis, pBi-NP, pMono-lucM and pRL (Table 1). Cells were harvested 48 h.p.t. and subjected to a luciferase assay. Firefly luciferase values were normalized to *Renilla* luciferase activity. As expected, the reporter construct with NCR from the M genomic segment showed by far the highest activity (normalized value of 3115.9) (Figure 10). Interestingly, NCRs from the PB1 segment were able to drive the second highest expression level (normalized value 2297.0) whereas the construct with the NS NCRs showed just 25% of the activity of the M NCR construct (normalized value of 814.7) (Figure 10).

### Expression of a whole antibody by the influenza replication machinery

**Figure 11:**
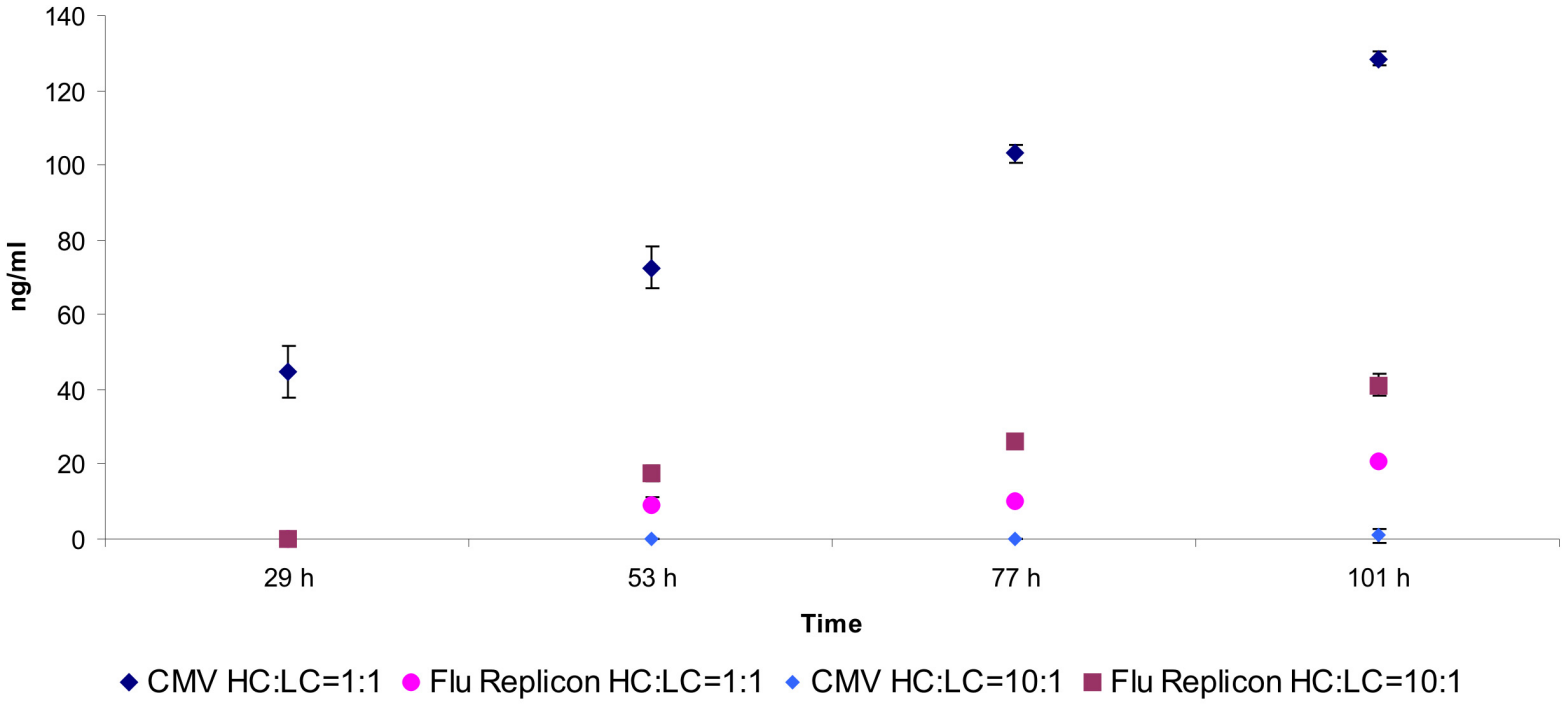
Expression of a the monoclonal anti-gp41 antibody 3D6. HEK-293 cells were either tranfected with pRC-LC and pRC-HC (CMV HC∶LC = 1∶1, CMV HC∶LC = 10∶1) or with the influenza replicon and pMono-LC and pMono-HC (Flu Replicon HC∶LC = 1∶1, Flu Replicon HC∶LC = 10∶1). Heavy chain to light chain ratios of 1∶1 and 10∶1 were tested. Antibody concentration in the culture supernatant was monitored for 101 hours by ELISA. Data represents arithmetic mean values and standard deviation.

In order to test the feasibility of the influenza A replicon system to express secreted proteins, we made constructs encoding the light and heavy chain of the human monoclonal anti-HIV-gp41 antibody 3D6. As positive control CMV promoter driven expression plasmids for heavy (pRC-HC) and light chain (pRC-LC) were used (Table 1). It is known that the heavy chain to light chain ratio of 1∶1 is in some cases suboptimal, therefore we decided to apply heavy chain to light chain ratios of 1∶1 and 10∶1. In the 10∶1 expression series we basically used the same amount of heavy chain coding plasmid as in the 1∶1 expressions but reduced the amount of light chain coding plasmid to a tenth of the heavy chain amount. Briefly, HEK-293 cells were transfected with pRC-HC and pRC-LC in ratios 1∶1 or 10∶1 or pTripolis, pBi-NP, pMono-HC and pMono-LC (pMono-HC and pMono-LC also in ratios 1∶1 and 10∶1) (Table 1). The antibody concentration in the culture supernatant was analyzed at four time points during a period of 101 hours by enzyme-linked immunosorbent assay (ELISA) (Figure 11). Interestingly, expression level of the pRC driven 1∶1 ratio samples (128.5 ng/ml) was eight times higher than their influenza replicon driven counterpart (20.9 ng/ml) at 101 h.p.t. (Figure 11). However, a completely different expression profile could be observed for the 10∶1 ratio expressions. The pRC driven 10∶1 expression produced only 0.9 ng/ml whereas the replicon system produced 41.1 ng/ml in this setting which is approximately 45 times as much antibody (Figure 11).

### Light chain expression

**Figure 12:**
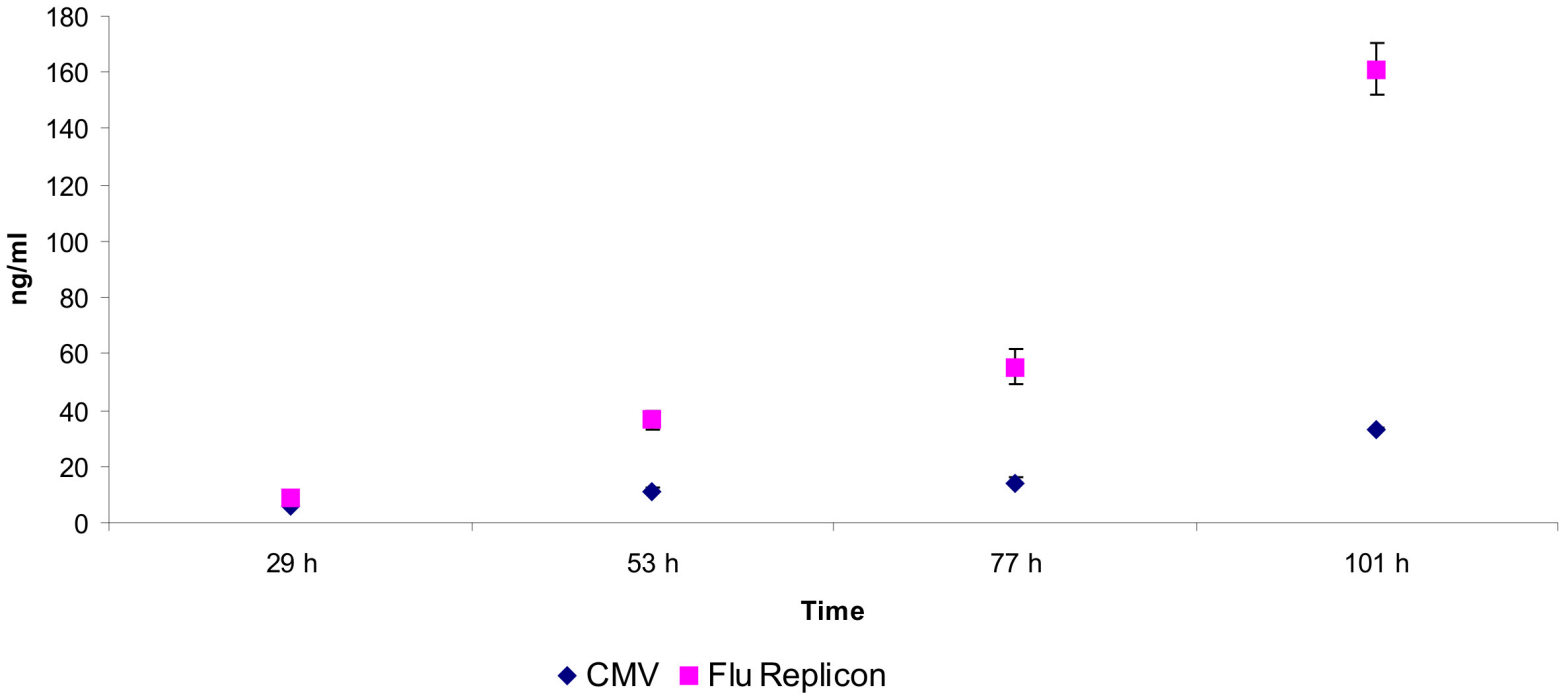
Light chain expression. HEK-293 cells were either tranfected with pRC-LC (CMV) or with the influenza replicon and pMono-LC (Flu Replicon). Light chain concentration in the culture supernatant was monitored for 101 hours by ELISA. Data represents arithmetic mean values and standard deviation.

In order to eliminate the observed ratio effect and to be able to determine single protein expression levels for a secreted protein we chose to express the 3D6 light chain only. HEK-293 cells were transfected with pRC-LC or the replicon system with pMono-LC. The light chain concentration in the culture supernatant was analyzed at four time points during a period of 101 hours by ELISA. Surprisingly, in this experimental set-up, the replicon driven expression showed higher expression levels than expression driven by pRC-LC (Figure 11). At the end point of the measurement period the culture supernatant of replicon transfected cells showed as much as 161.2 ng/ml light chain which is approximately five times the amount that was achieved by the pRC-LC control (Figure 12) (32.8 ng/ml light chain).

## Discussion

In this study we investigated the feasibility of influenza A virus replicons for transient expression of recombinant proteins in mammalian cells. In a first experiment we compared the transfection efficiencies of the plasmid based influenza replicon system expressing EGFP and the expression vector pEGFP-N1 (CMV). The replicon based system showed a delayed expression profile compared to the pEGFP-N1 driven expression, transfection efficiency of the influenza replicon system was approximately 50% lower (Figure 3). We hypothesize that there are two main reasons for the reduced transfection efficiency of the replicon based system. The CMV driven system is based on one plasmid whereas the replicon system for EGFP expression is based on three different plasmids, decreasing the statistical probability to have all components present in the same cell. Additionally, pEGFP-N1 is 4733 bp in size whereas the plasmids used for the replicon system are 3082, 5116 and 17035 bp in size. It is well known that the transfection efficiency decreases dramatically with increased plasmid size [22]. In addition, a certain degree of cytotoxicity of the influenza replicon activity or its components and therefore a negative influence on long-term expression cannot be ruled out. As shown by FACS analysis, cells transfected with the replicon system showed two distinct populations of cells (Figure 5). One population remained totally negative whereas a second, quite large population (19.4%) was highly EGFP positive. However, the overall expression level was only between 13% and 27% of the levels expressed by the CMV promoter (Figure 6). However, when compared to the SV40 promoter, the influenza replicon system showed approximately 100 fold higher activity (Figure 7).

We showed that the activity of the influenza replicon system is tightly restricted to primate cells. Almost no activity was found in a canine and a rodent cell line and full activity was detected in human HEK-293 cells (Figure 8) and has also been shown previously for Vero cells [23]. The RNA polymerase I promoter used in this expression system for the initial transcription of vRNA is derived from human non-transcribed spacer 5′ rDNA regions. RNA polymerase I promoters, which are used by the cell for rRNA transcription, are known to be species specific [24]. However, by changing to a homologous RNA polymerase I promoter the replicon system could be adapted to virtually any cell line. Similar systems have been reported for canine and avian cells [25], [26].

Temperature is known to have a strong influence on influenza A virus replication. For example, fever is involved in the defense of the human body against influenza virus infections. On the other hand, for recombinant protein expression lower temperatures have some advantages such as lower energy consumption for heating in large scale production, proper protein folding and less cell stress [27]. We therefore, tested replicon activity at 27°, 37° and 40°C. While pcDNA-luc (CMV) and pGL3-control (SV40) driven expression was shown to be temperature independent, the influenza replicon system turned out to be highly temperature sensitive. Replicon based expression completely disappeared at 27°C. This finding becomes relevant for expression of influenza polymerases and ribonucleoprotein complexes in insect cells, which are usually grown at 27°C or below. The activity of the replicon also decreased to about 25% at 40°C as compared to the full activity at 37°C (Figure 9).

Initially, our reporter and gene of interest constructs for generation of vRNA were flanked by 5′ and 3′ non-coding regions of the NS genomic segment. In order to find out if expression can be increased by the use of NCRs from other genomic segments, we prepared luciferase reporter constructs flanked by 5′ and 3′ regions of the M and PB1 genomic segment. By changing the flanking regions we were able to increase expression 2.8-fold in case of the PB1 NCRs and 3.8-fold in case of the M NCRs (Figure 10). In contrast to the M NCR, which is only present on the reporter vRNA, the PB1 NCR is also present on the PB1 vRNA derived from plasmid pTripolis. This has to be considered since replicational competition between two vRNA species with the same NCR cannot be ruled out. It is also notable, that NCRs from the NS genomic segment, which encodes for non-structural proteins, showed the lowest activity whereas NCRs from the M genomic segment, coding for the most abundant structural protein M1, showed the highest activity. The activity of the NCRs derived from the PB1 genomic segment lied in between.

To test whether the replicon system is suitable as an expression system for secreted proteins the anti-HIV-gp41 antibody 3D6 was expressed applying different ratios of heavy and light chain gene dosage. In the 1∶1 ratio series the pRC (CMV) driven system produced a six fold higher antibody concentration (128.5 ng/ml) than the replicon system (Figure 11) (20.9 ng/ml, 101 h.p.t.). Interestingly, in the 10∶1 series (amount of light chain vector was reduced to a tenth of the heavy chain vector concentration) we found 41.1 ng/ml (101 h.p.t.) in the supernatant of the replicon driven system which was approximately twice the amount found in the 1∶1 series (Figure 11). The CMV promoter system produced only small amounts (0.9 ng/ml) of antibody in this experimental setting (Figure 11). The optimal ratio between heavy chain and light chain vector for the CMV driven constructs was found somewhere in between 1∶1 and 10∶1, at least in CHO cells [28]. Both, heavy chain and light chain sequences used for vRNA transcription were flanked by NCRs of the influenza NS genomic segment. The vRNA comprising the heavy chain sequence is approximately twice as long as the vRNA hosting the light chain. Therefore, we speculate that there was has been a replicational competition between both vRNA species. The shorter vRNA was probably amplified at a higher rate and to a higher number. This would explain why an initial excess of heavy chain vector was beneficial for the expression of the antibody. This effect was definitely not observed for pRC driven expression, where reduction of light chain coding plasmid led to an almost complete shut down of antibody expression. In order to eliminate this competition effect and to be able to directly compare expression levels of secreted proteins, we investigated the behavior when only light chain was present. In this experimental set-up the replicon system was able to drive expression far more efficiently than the pRC control (Figure 12). Light chain concentrations in the supernatant were fivefold higher for the replicon system (161.2 ng/ml) than for the CMV driven expression 101 h.p.t. (Figure 12). Interestingly, influenza replicon driven light chain expression exceeded CMV driven light chain expression while for EGFP and firefly luciferase expression the CMV promoter showed higher activity than the replicon. This might be explained by the fact that, in contrast to EGFP and firefly luciferase, the antibody light chain is transported through the Golgi and secreted to the supernatant. The folding and secretion pathway is the major bottle neck in secreted protein production, strongly depending on the individual protein and the metabolic burden by over-expression. We speculate that influenza replicon transcription activity rates may meet the optimal transcription rates for accurate folding and secretion, while these pathways are overloaded by CMV promoter activity. Measuring the actual transcription rates might help to elucidate this phenomenon.

In conclusion, we demonstrated that expression efficiencies of the influenza replicon were comparable with a widely used transient CMV expression system. Luciferase assays were suitable to evaluate temperature sensitivity of the replicon system, showing complete shut-down of activity at 27°C. Additionally, we demonstrated that the choice of flanking NCRs has strong influence on the overall expression level, and have to be chosen accordingly. We could further show that the influenza replicon is feasible for high level expression of complex secreted proteins in human cell lines. As has been shown for a human antibody light chain, for some proteins the product yield can be increased substantially above the levels of CMV promoter driven expression.

## Materials and Methods

### Cells and Viruses

The human cell line HEK-293 (ATCC # CRL-1573) was cultured in RPMI 1640 medium (Biochrom, Berlin, Germany) supplemented with 10% fetal calf serum (FCS) (PAA, Pasching, Austria). The canine cell line MDCK (ATCC # CCL-34), the african green monkey cell line Vero (ATCC # CCL-81), and the Chinese hamster cell line CHO-K1 (ATCC # CCL-61) were cultured in serumfree DMEM/Ham's F12 medium (Biochrom, Berlin, Germany). Influenza strain A/Hiroshima/52/2005 (H3N2, Gene Bank Entries EU285583, EU283414, EU597800–EU5978005) was grown on Vero cells in serum free DMEM/Ham's F12 supplemented with antibiotics and trypsin (Polymun, Vienna, Austria).

### Vectors and Vector Construction

Bidirectional transcription cassettes for influenza genes were designed as described by Hoffmann and Neumann et al [11], [29]. A CMV promoter (derived from pCI, Promega, Madison, USA) in sense direction drives mRNA transcription which is terminated by a SV40 polyA signal. Minus-sense single strand (−ss) RNA transcription is mediated by a human RNA polymerase I dependent promoter fragment (RNA polymerase I promoter) in antisense direction and terminated by a mouse RNA polymerase I dependent terminator (Figure 2). Between RNA polymerase I promoter and terminator two *Bsm*BI restriction sites are located as described by Neumann et al [30]. The bidirectional cassette was synthesized by Geneart (Regensburg, Germany), amplified by PCR with primers carrying *Bam*HI and *Not*I restriction sites and cloned into a modified pUC19 (NEB, Ipswitch, USA) vector lacking the lacZ gene resulting in pBi (Figure 2). The core of the transcription cassette consisting of the RNA polymerase promoter and terminator was also amplified by PCR using primers carrying *Bam*HI and *Not*I restriction sites and cloned into a modified pUC19 vector (NEB) lacking the lacZ gene resulting in pMono.

Complementary DNA (cDNA) fragments of NP (EU597803.1), PA (EU597802.1), PB1 (EU597801.1), PB2 (EU597800.1) from A/Hiroshima/52/2005 were obtained by TRIZOL™ extraction and reverse transcription of Vero infection supernatant and cloned into pBi by the use of *Bsm*BI restriction enzyme or by the In-Fusion PCR Cloning System (Clontech, Mountain View, USA) resulting in pBi-NP, pBi-PB1, pBi-PB2 and pBi-PA.

The pTripolis plasmid which hosts bidirectional expression cassettes for all three influenza polymerases was cloned as follows: pBi-PB2 was digested by *Not*I and *Bam*HI and the bidirectional transcription cassette was cloned in a *Not*I and *Bam*HI digested pTriEx1.1 plasmid (Merck, Darmstadt, Germany) resulting in p3X-PB2. pBi-PB1 was digested by *Not*I and *Bam*HI and the bidirectional transcription cassette was cloned in a *Not*I and *Bam*HI digested pFastBac Dual plasmid (Invitrogen, Carlsbad, USA) resulting in pFB-PB1. pBi-PA was digested by *Nhe*I and *Pci*I and the bidirectional transcription cassette was cloned in a *Nhe*I and *Pci*I digested pFastBac Dual (Invitrogen) resulting in pFB-PA. Vector p3X-PB2 was then cut by *Pml*I and *Pac*I restriction endonucleases and the PB2 cassette was cloned into a *Pac*I and *Bst*Z17I digested pFB-PB1 resulting in pFB-PB1-PB2. Vector pFB-PA was finally cut with *Sph*I and *Pac*I restriction endonucleases and the PA cassette was cloned into the *Sph*I and *Pac*I digested pFB-PB1-PB2 resulting in pTripolis (Figure 1).

The plasmid pEGFP-N1 (Clontech) was used as control vector in FACS experiments. The CMV driven control vector for the luciferase experiment was constructed by *Bam*HI and *Xba*I excision of the firefly luciferase gene from pGL3-Control (Promega) and cloning into a *Bgl*II and *Xba*I restricted pcDNA3 (Invitrogen) resulting in pcDNA-luc. Additionally, the original pGL3 was used as SV40 promoter control.

The plasmids pRC-HC (heavy chain) and pRC-LC (light chain) encode heavy and light chain from the human monoclonal anti-HIV-gp41 antibody 3D6 under the control of a CMV promoter as described by Rüker et al [17]. The heavy chain nucleotide sequence was altered on genetic level by site directed mutagenesis to remove the *Bsm*BI restriction site. The amino acid sequence was not altered.

Heavy chain and light chain of 3D6, EGFP-N1 and firefly luciferase were amplified by PCR with primers containing 5′ and 3′ non-coding regions of the NS genomic segment of A/Hiroshima/52/2005 and cloned into pMono using *Bsm*BI restriction endonuclease resulting in pMono-HC, pMono-LC, pMono-EGFP and pMono-lucNS. The firefly luciferase gene was further amplified by PCR with primers containing 5′ and 3′ non-coding regions of the PB1 or M genomic segment of A/Hiroshima/52/2005 and cloned into pMono using *Bsm*BI restriction endonuclease resulting in pMono-lucPB1 and pMono-lucM. Sequences were confirmed by Sanger sequencing.

The plasmid pRL-SV40 (Promega, Madison, USA) which encodes the *Renilla* luciferase was used as internal control for luciferase experiments. All plasmids used were prepared from over night cultures of *E. coli* K12 JM109 (NEB) with a NucleoBond Xtra Midi EF kit (Machery-Nagel, Düren, Germany). Primer sequences are available on request.

### Transfection

Transfections were carried out with an Amaxa Nucleofector I device (Lonza, Basel Switzerland). The day before transfection, cells were split 1∶3 to obtain cells in log phase. Briefly, HEK-293 and MDCK cells were washed with PBS and treated with trypsin (PAA) to detach them from flask surfaces. After detachment, cells were diluted in PBS containing trypsin inhibitor (Polymun). CHO cells were grown in suspension and were harvested directly. Cells were counted and aliquots containing 4×10^6^ cells were centrifuged at 1000 g for 5 minutes. Supernatant was removed and 4×10^6^ cells per transfection were used for Nucleofection with a Nucleofector Kit V as described by the manufacturer (HEK-293 program A23, MDCK program A24 and CHO program H14). After transfection cells were resuspended in pre-warmed medium containing 10% FCS and antibiotics. Cells transformed for antibody or light chain expression were directly resuspended in 2.5 ml medium in 6-well plates whereas cells for other experiments were resuspended in 10 ml medium and aliquots of 1 ml were seeded in 12-well plates.

Cells dedicated to FACS analysis were transfected with 9 µg of pEGFP-N1 or 3 µg of pTripolis, pBi-NP and pMono-EGFP (9 µg total DNA) (Table 1). Untransfected HEK-293 cells served as negative control.

Cells dedicated to luciferase assays were transfected with 9 µg pcDNA-luc or 9 µg pGL3-control or 3 µg of pTripolis, pBi-NP and pMono-lucNS (9 µg total DNA) or 3 µg of pTripolis, pBi-NP and pMono-lucPB1 (9 µg total DNA) or 3 µg of pTripolis, pBi-NP and pMono-lucM (9 µg total DNA) or 3 µg of pBi-NP and pMono-lucNS (6 µg total DNA) as negative control. Additionally 1 µg of pRL was added to each of the transfections dedicated to luciferase assays. In case of MDCK and CHO-K1 cells, untransfected cells were used as negative control.

Cells dedicated to CMV driven antibody or light chain expression were either transfected with 4.5 µg pRC-HC and 4.5 µg pRC-LC (9 µg total DNA) or 4.5µg of pRC-HC and 0.45 µg of pRC-LC (4.95 µg total DNA) or 9 µg of pRC-LC. Cells dedicated to influenza replicon driven antibody or light chain expression were transfected with 3 µg of pTripolis, pBi-NP, pMono-HC and pMono-LC (12 µg total DNA) or 3 µg of pTripolis, pBi-NP, pMono-HC and 0.3 µg of pMono-LC (9.3 µg total DNA) or 3 µg of pTripolis, pBi-NP and pMono-LC (9 µg total DNA) (Table 1). Untransfected HEK-293 cells served as negative control.

Transfected cells dedicated to temperature experiments were allowed to settle for 4 hours at 37°C and were then transferred either to 40°C or 27°C incubators. All transfection experiments were performed in duplicates.

### FACS

FACS experiments were conducted to determine the proportion of GFP expressing cells over a period of 101 hours. HEK-293 cells were transfected, seeded into 12-well plates as described and analyzed 8, 29, 53, 77 and 101 hours post transfection. Non-transfected HEK-293 cells were used as negative control. Supernatant was removed, cells were resuspended in 300 µl PBS and 10 000 cells were analyzed using a BD FACS Canto II device. FACS experiments were performed in technical duplicates resulting in four measuring points taking the biological duplicates into account. Data represents arithmetic mean values and standard deviation.

### Luciferase Assay

Briefly, the cell supernatant was removed and the cells were resuspended in 300 µl PBS. Aliquots of 50 µl (approximately 6.6×10^4^ cells) were taken from the cell suspension and were transferred into black 96-well plates. Samples were analysed regarding firefly and *Renilla* luciferase activity using the Dual Glo Luciferase Assay System (Promega, Madison, USA) according to the manufacturer's recommendations. Readouts were performed in a Tecan infinite M1000 reader (Tecan, Männedorf, Switzerland) using i-control 1.6 software (readout settings for both, firefly and *Renilla* luciferase: integration time 10000, attenuation: none, settle time 0). Luciferase assays were performed in technical duplicates resulting in four measuring points taking the biological duplicates into account. Firefly luciferase values were normalized to *Renilla* luciferase activity. In case of luciferase time curve experiments absolute firefly values were used due to the time dependence of the *Renilla* expression. Data represents arithmetic mean values and standard deviation.

### Antibody and light chain quantification

HEK-293 cells were transfected with full antibody or light chain only expressing constructs as described, seeded into 6-well plates and samples from supernatant were taken 29, 53, 77 and 101 hours post transfection. The amount of 3D6 antibody or 3D6 light chain was determined by enzyme-linked immunosorbent assay (ELISA). 96-well microtiter plates (Nunc-Immuno Plate Maxisorp) were coated with anti-human κ light chain antibody (Sigma K3502, diluted 1∶1000, goat origin) in case of light chain detection or with anti-human IgG γ-chain specific antibody (Sigma I3382, diluted 1∶1000, goat origin) in case of whole antibody detection in coating buffer over night at 4°C. Plates were washed with PBS containing 0.005% Tween 20 and incubated with serial two-fold dilutions of 1∶3 or 1∶5 pre-diluted supernatant samples (room temperature, 2 h). Afterwards plates were washed three times and incubated with peroxidase conjugated anti-human kappa light chain antibody (Sigma A7164, diluted 1∶2000, goat origin) in PBS containing 1% BSA and 0.005% Tween 20 (room temperature, 1h). Unbound antibody was removed and plates were washed. Samples were incubated with substrate (o-phenylene diamine and H_2_O_2_), reaction was stopped using H_2_SO_4_ and the colorimetric change was measured at 492 nm. Samples were quantified relative to a 3D6 antibody standard of known concentration. In case of light chain detection, the same 3D6 antibody standard was used, a concentration of one third of the whole antibody was assumed because the light chain represents approximately one third of the mass of a whole antibody. ELISA assays were performed in technical duplicates resulting in four measuring points taking the biological duplicates into account. Data represents arithmetic mean values and standard deviation.
